# Successful intragastric suture repair using endoscopy for refractory ischemic stomach perforation: A case report

**DOI:** 10.1016/j.amsu.2021.102212

**Published:** 2021-03-08

**Authors:** Hiroki Kajioka, Atsushi Muraoka

**Affiliations:** Department of Surgery, Kagawa Rosai Hospital, 3-3-1 Joto-cho, Marugame-shi, Kagawa, 763-8502, Japan

**Keywords:** Intragastric suture repair, Endoscopy, DP-CAR, Refractory ischemic stomach perforation, Pancreatic cancer

## Abstract

**Introduction:**

Ischemic stomach perforation and bleeding are major complications after distal pancreatectomy with en bloc celiac axis resection (DP-CAR) for locally advanced pancreatic cancer. Although there are some treatment options for ischemic gastric events, we need to discuss the optimal treatment based on the patient's general condition and history.

**Case presentation:**

A 76-year-old woman with advanced pancreatic cancer underwent DP-CAR with the reconstruction of the common hepatic artery-celiac artery. She presented with a high fever and melena at 13 days and twenty-nine days after the operation, respectively. Contrast-enhanced computed tomography (CECT) demonstrated ischemic stomach perforation, which was localized. Although nonsurgical treatments, including endoscopic clipping and proton-pump inhibitor administration, were attempted, her symptoms were not relieved. Therefore, we performed intragastric suture repair using oral endoscopy (ISE) for gastric perforation. Although she presented with surgical site infection and a catheter-related blood stream infection after ISE, she was discharged 140 days after the first operation.

**Clinical discussion:**

Ischemic gastric events following DP-CAR can be treated with non-surgical and surgical approaches. In this case, non-surgical management could not improve the patient's gastric complications, and she had to undergo surgery. Given the patient's condition, ISE was an indication for this complication and was, thus, performed among surgical procedures, resulting in the alleviation of the complication. Using ISE may provide safe and less invasive surgery.

**Conclusion:**

This is the first case of ISE for use in refractory ischemic stomach perforation following DP-CAR. ISE can be a useful and minimally invasive surgical procedure.

## Highlights

• DP-CAR sometimes induces gastric perforation due to insufficient gastric bloodstream.
• LGA preservation may not prevent gastric ischemic events.
• Intragastric suture repair using oral endoscopy (ISE) can be minimally invasive.
• ISE can be an indication for refractory bleeding and perforation.
• ISE is contraindicated in cases where a transverse colon is just over the stomach.

## 1 Introduction

Distal pancreatectomy with en bloc celiac axis resection (DP-CAR) is performed for locally advanced pancreatic carcinoma involving the common hepatic artery (CHA) and splenic artery (SpA). Although DP-CAR dramatically increases tumor resectability [1], the associated postoperative morbidity rate is high [2]. Among postoperative complications, there are ischemic gastric events that are sometimes fatal. There are some treatment options for ischemic gastric events, including surgical or nonsurgical procedures. However, the indications for treatment have been controversial and there is no gold standard for ischemic gastropathy post-DP-CAR. We need to select the optimal treatment based on patient's general condition and history. Here, we report a case of ischemic stomach perforation following DP-CAR rescued with intragastric suture repair using oral endoscopy (ISE). This case report has been reported in line with the SCARE Criteria [3].

## 2 Presentation of case

**Fig. 1:**
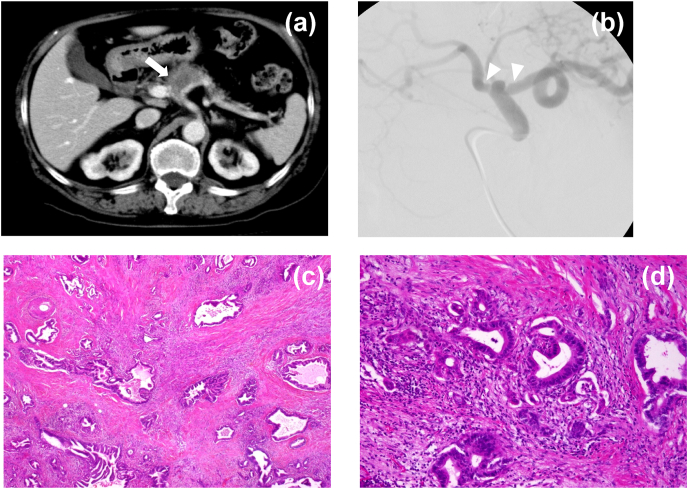
Contrast-enhanced computed tomography (CECT) showing a hypovascular tumor in the pancreatic body (arrow) (a). Angiography showing encasement of both splenic artery and common hepatic artery (arrowhead) (b). Histological findings showed invasive ductal adenocarcinoma with a dense fibrotic stroma (c: × 20, d: × 100).

A 77-year-old Japanese woman was referred to our hospital for further examination of the pancreatic mass. She presented with epigastric tenderness. She had no relevant medical history, took no medications, and had no allergies. Similarly, she also had no relevant family history and was not a habitual drinker or smoker. Contrast-enhanced CT (CECT) revealed a 6 × 2cm hypo-vascular mass in the pancreatic body involving the CHA and SpA (Fig. 1a). Similarly, angiography showed that the pancreatic mass invaded the CHA and SpA (Fig. 1b). She was preoperatively diagnosed with pancreatic cancer involving the CHA and SpA, which was an indication for DP-CAR. Intraoperative findings revealed a nodule in the pancreatic body, invading both the CHA and SpA without any peritoneal dissemination. The CHA clamp test performed showed that the CHA flow decreased from 24 mL/s to 9 mL/s. Therefore, we reconstructed the CHA-celiac artery (CA) to prevent gastric and hepatic ischemia. However, the reconstruction was unsuccessful. She underwent planned DP-CAR without reconstructing the CHA-CA and preserving the right epiploic artery and gastric artery. Histopathological findings showed 6 × 2.5 × 1.5 cm well-differentiated invasive ductal carcinoma (Fig. 1c and d) with a lymph node metastasis involving the neuroplexus of CHA and SpA without direct vascular invasion, stageⅡB (T3N1aM0).

**Fig. 2:**
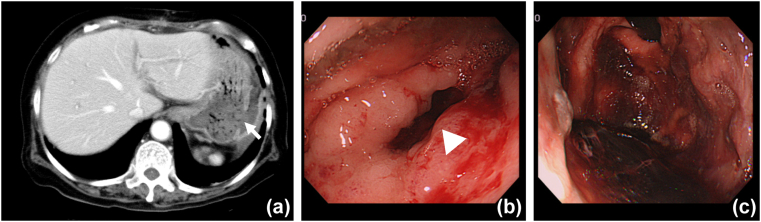
CECT showing gastric perforation (arrow) surrounding abscess (arrow) (a). Endoscopic findings showing gastric wall cavity (arrow head) (b) and blood clots (c).

**Fig. 3:**
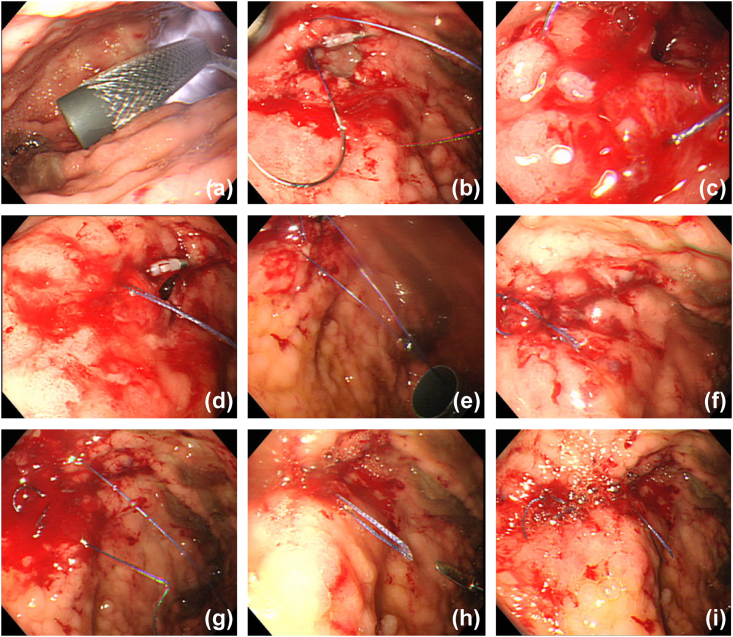
Surgical procedure. (a) A 12-mm port was directly into the gastric lumen under direct vision from endoscopy. (b–h) Three interrupted stitches of full-thickness with an extracorporeal knot using the laparoscopic knot pusher. (i) The oral endoscopic findings after gastric wall repair.

She was discharged from the intensive care unit two days postoperatively. However, she suddenly presented with a high fever of 101.2° at 13 days post-operation. Physical findings demonstrated rebound epigastric tenderness over the left upper quadrant. CT revealed two abscesses around the greater curvature of the fundus and remnant pancreatic stump. The patient was treated with antibiotics because her abscess was localized, and 20mg of a proton-pump inhibitor continued to be intravenously administered twice a day, immediately after the operation. Although the high fever was relieved in three days, she started to present with frequent melena twenty-nine days post-operatively. CECT showed gastric perforation, which was localized, surrounded by an abscess (Fig. 2a). Endoscopic findings demonstrated a gastric wall cavity in the upper gastric body (Fig. 2b) with blood clots (Fig. 2c). Although non-surgical treatments, including endoscopic clipping and proton-pump inhibitor administration, were attempted, the complication was not alleviated. Her general status was poor, and postoperative adhesions were concerning. Therefore, ISE was planned and performed by our chief surgeon at district general hospital. The patient was placed in the supine position under general anesthesia. First, endoscopy was introduced into the stomach, followed by the inflation of the stomach using air insufflation. Thereafter, a 12-mm port was directly inserted into the gastric antrum, from the left hypochondrium, through the abdominal wall to the anterior gastric wall under oral endoscopic observation. Afterward, a needle holder and 3–0 absorbent suture were inserted into the stomach through the 12 mm port, followed by interrupted sutures of full-thickness with extracorporeal ligation. We repaired the gastric perforation using a three-interrupted suture (Fig. 3). Finally, no bleeding was confirmed in the repaired site under endoscopy. The stomach's entry hole was closed with a double-layer interrupted suture, and the abdominal port site was equally closed without a drainage tube. The operating time was 1 hour 50 minutes, blood loss was 30mL, and the procedure was performed as planned. After ISE, blood transfusions was performed, and some antibiotics were administered to treat catheter-related bloodstream infection due to the long-term placement of a central venous catheter and surgical site infection; she was relieved from refractory ischemic stomach perforation with bleeding, resulting in discharge 140 days post-DP-CAR. After discharge, oral administration of 30 mg a proton-pump inhibitor once a day was continued in compliance with our instruction and adjuvant chemotherapy was started. However, she died 870 days after DP-CAR due to pancreatic cancer progression.

## 3 Discussion

Pancreatic cancer is the seventh leading cause of cancer-related deaths worldwide [4]. It is so lethal that there are no symptoms until cancer progression. In addition, this cancer easily invades the surrounding organs, nerves, and vessels. Therefore, most cancer cases are diagnosed at an advanced stage. Given the silent and aggressive features of this cancer, radical surgical procedures are sometimes indispensable to cure. Among them, DP-CAR can be a proper procedure for locally advanced pancreatic body cancer [5] which involves both SpA and CHA. However, DP-CAR has a high mortality rate ranging between 2% and 16.7%, with a high morbidity rate of 49.4% [2]. Among postoperative complications, gastric ischemic events sometimes occur, including the development of gastric ulcers and perforation. A systematic review of 18 articles, reported that the incidence of gastric ischemic events was 12.87% [2].

Additionally, a retrospective and prospective study of 77 and 18 patients, respectively, demonstrated that almost all DP-CAR patients showed various mucosal damages (redness, erosion, edema, and congestion) [6]. These events resulted from trouble with the gastric blood circulation. Therefore, two methods have been attempted: preservation of the left gastric artery (LGA) and preoperative LGA embolization. A previous retrospective study of fifty patients demonstrated that LGA resection in DP-CAR was a risk factor for ischemic gastric events [7]. Additionally, some institutes performed preoperative LGA embolization to prevent ischemic gastric events. The incidence rates of gastric ischemia with or without preoperative embolization were 10.74% and 14.38%, respectively [2]. Because these techniques' effectiveness may be limited, if attempted, this technique might not have prevented gastric ischemia in the present case.

Management of gastric ulcer and perforation with and without bleeding involves surgical and non-surgical treatment. Non-surgical treatment includes fasting, administration of proton-pump inhibitors and antibiotics, and endoscopic treatment. Regarding gastric ulcer and perforation with bleeding, a systematic review showed that endoscopic clipping is a safe and effective technique [8]. However, endoscopic clipping was not be effective in this case due to the large perforation size. Therefore, we selected a surgical procedure. The surgical procedure includes partial gastric resection and suture repair with or without omental implantation. A retrospective study of 815 patients demonstrated that surgical procedures are indications for intractable bleeding after non-surgical therapy [9]. However, in this case, we were concerned about the strong adhesion between the abdominal wall and organ due to a laparotomy history and the elapsed time after DP-CAR. Given the re-operation, there were some possibilities of prolonged operating time, massive bleeding, and other organ injuries, leading to general deterioration. Therefore, ISE was performed in this case. In addition, after ISE, she did not require additional surgical and endoscopic procedures. Therefore, ISE was a feasible and effective procedure.

Percutaneous endoscopic intragastric surgery, a procedure for treating gastrointestinal stromal tumor, was first reported by Kanehira et al. [10] and was considered applicable to this case.

ISE is a safe and minimally invasive method, which could close the gastric perforation site completely and effectively stop gastric bleeding. Although ISE requires a single incision, no significant abdominal incision is needed. Given the strong abdominal adhesion, ISE led to reduced operating time and blood transfusions. Compared with open laparotomy with gastric resection, ISE was a minimally invasive treatment for refractory ischemic stomach perforation and bleeding. Thus, ISE may be a feasible method for refractory gastric bleeding and perforation with localized abscess. In this case, ISE was performed under general anesthesia; however, a case that is in critical condition, or cannot tolerate general anesthesia, might be undertaken under local anesthesia.

There are some limitations and disadvantages to ISE. First, direct port-placement poses a risk of injury to the surrounding organs. For example, when a transverse colon is placed over the stomach, ports cannot be inserted directly. Therefore, we should pay meticulous attention to the findings of CT images before ISE. Second, if intraperitoneal contamination is all over the peritoneal cavity, this procedure might not be sufficient. In such cases, laparotomy is required to remove intraperitoneal contamination, wash the peritoneal cavity, and place the drain tube. Third, the entry hole in the stomach might cause another gastric ulcer and perforation with bleeding. To prevent this complication, we inserted the port through the gastric antrum, which had better blood circulation.

## 4 Conclusion

In conclusion, we report a case of ISE for refractory ischemic stomach perforation and bleeding after DP-CAR. Although ISE has some limitations and disadvantages, it can be a useful approach for gastric perforation and bleeding with localized intraperitoneal contamination following DP-CAR.

### 4.1 Patient perspective

The procedure of surgery was explained to the patient with all advantage and possible complications. She agreed to the procedure and provided informed consent.

### 4.2 Informed consent

Written informed consent was obtained from the patient for publication of this case report and accompanying images. A copy of the written consent is available for review by the Editor-in-Chief of this journal on request.

## Provenance and peer review

Not commissioned, externally peer-review.

## Ethical approval

The requirement for ethical approval was waived by the Institutional Review Board because this was case report.

## Sources of funding

The authors have no sponsors.

## Author contribution

Hiroki Kajioka drafted the manuscript. Atsushi Muraoka performed the operation and reviewed the manuscript.

## Trial registry number

Name of the registry: not applicable.

Unique identifying number or registration ID: not applicable.

Hyperlink to your specific registration (must be publicly accessible and will be checked): not applicable.

## Guarantor

Hiroki Kajioka.

## Declaration of competing interest

All authors have nothing to disclose.
